# Identifying Habitat Elements from Bird Images Using Deep Convolutional Neural Networks

**DOI:** 10.3390/ani11051263

**Published:** 2021-04-27

**Authors:** Zhaojun Wang, Jiangning Wang, Congtian Lin, Yan Han, Zhaosheng Wang, Liqiang Ji

**Affiliations:** 1 Key Laboratory of Animal Ecology and Conservation Biology, Institute of Zoology, Chinese Academy of Sciences, Beijing 100101, China; wangzhaojun@ioz.ac.cn (Z.W.); wangjn@ioz.ac.cn (J.W.); linct@ioz.ac.cn (C.L.); hanyan@ioz.ac.cn (Y.H.); 2 College of Resources and Environment, University of Chinese Academy of Sciences, Beijing 100101, China; 3 National Ecosystem Science Data Center, Key Laboratory of Ecosystem Network Observation and Modeling, Institute of Geographic Sciences and Natural Resources Research, Chinese Academy of Sciences, Beijing 100101, China; wangzs@igsnrr.ac.cn

**Keywords:** bird images, deep convolutional neural networks, habitat elements

## Abstract

**Simple Summary:**

To assist researchers in processing large amounts of bird image data, many algorithms have been proposed, but almost all of them aim at solving the problems of bird identification and counting. We turn our attention to the recognition of habitat elements in bird images, which will help with automatically extracting habitat information from such images. To achieve this goal, we formed a dataset and implemented our proposed method with four kinds of deep convolutional neural networks, and the recognition rate reached a minimum of 89.48% and a maximum of 95.52%. The use of this method will supplement the extraction of bird image information and promote the study of the relationships between birds and habitat elements.

**Abstract:**

With the rapid development of digital technology, bird images have become an important part of ornithology research data. However, due to the rapid growth of bird image data, it has become a major challenge to effectively process such a large amount of data. In recent years, deep convolutional neural networks (DCNNs) have shown great potential and effectiveness in a variety of tasks regarding the automatic processing of bird images. However, no research has been conducted on the recognition of habitat elements in bird images, which is of great help when extracting habitat information from bird images. Here, we demonstrate the recognition of habitat elements using four DCNN models trained end-to-end directly based on images. To carry out this research, an image database called Habitat Elements of Bird Images (HEOBs-10) and composed of 10 categories of habitat elements was built, making future benchmarks and evaluations possible. Experiments showed that good results can be obtained by all the tested models. ResNet-152-based models yielded the best test accuracy rate (95.52%); the AlexNet-based model yielded the lowest test accuracy rate (89.48%). We conclude that DCNNs could be efficient and useful for automatically identifying habitat elements from bird images, and we believe that the practical application of this technology will be helpful for studying the relationships between birds and habitat elements.

## 1. Introduction

Monitoring the populations and habitats of wild animals and plants is not only very important for protecting biodiversity but also closely related to human survival and development. Because of their wide distribution, great mobility, and high sensitivity to environmental changes [1], birds have naturally become extremely important groups for monitoring. Time-lapse videos [2,3], camera traps [4,5,6,7,8,9], and unmanned aerial vehicle (UAV) [10,11,12] aerial photographs are widely used in bird monitoring. The advantages of using these devices are high security, long-term use, uninterrupted monitoring, minimal interference with birds, and surveying in areas where humans cannot easily stay for long periods of time. In addition, the captured images can be stored conveniently. A large number of bird images can be easily obtained by a professional ornithologist or birdwatcher using a variety of image acquisition devices (e.g., regular cameras or smartphones). As an important part of bird monitoring data, bird images are of great significance in bird monitoring activities. Proverbially, a picture says more than a thousand words, and bird images can record the appearances, behaviours, population characteristics, and habitat elements of the observed birds directly and quickly. In particular, by analysing the habitat elements in bird images, we can find patterns between birds and habitat elements (e.g., preferences for pine foliage or trunks for foraging or nesting [13]), and these patterns are helpful for understanding bird behaviour and monitoring the impacts of environmental changes on birds. However, the collection of bird images from around the world is growing so rapidly that it has greatly outpaced the abilities of image analysis tools [14]. It is conceivable that the cost of manually extracting habitat elements from a large amount of image data for analysis purposes is extremely high or even unfeasible. Unfortunately, no algorithm that can automatically identify habitat elements in bird images has been proposed, which has prevented scientists from using bird images to carry out relevant research.

In recent years, deep learning [15,16] has made remarkable achievements [17] in computer vision [18,19]. Deep learning has yielded great improvements in object detection [20], object recognition [21], scene recognition [22], image segmentation [23], and other tasks. Deep learning techniques, such as deep convolutional neural networks (DCNNs), have also attracted the attention of ecologists. DCNNs can automatically learn from data. Taking image classification tasks as an example, DCNNs can automatically learn features for classification from a large number of input images, without relying on human domain knowledge. This remarkable advantage makes researchers only need to collect corresponding data according to the target when using this technology, instead of making various attempts for feature selection and extraction. To assist ecologists and zoologists in rapidly and effectively processing large-scale bird image data, computer vision research has long dealt with bird image analysis-related problems, such as bird detection [24], the counting of crowded birds [25,26], fine-grained classification [27,28,29,30] of birds, and even individual recognition with small birds [31], using DCNNs. DCNNs have achieved surprising results in these tasks.

Such studies are very helpful for performing bird monitoring research. However, few studies have been conducted on the recognition of habitat elements, which are very important for studying the relationships between birds and the environment. Habitat elements are generally located in image backgrounds. In studies by [32,33], an algorithm was proposed that can identify whether animals (including birds) are present in an image. This algorithm can quickly divide animal images into two categories; this has been of great help to researchers but still fails to meet their actual needs. When studying the relationships between birds and habitat elements, such as those between birds and wires [34,35], even though researchers can collect a large amount of relevant image data using various image acquisition equipment, they must use artificial methods in the data processing stage and analyse the obtained images one by one or frame by frame. Such a process requires considerable manpower and time; the manual processing method is only suitable for a relatively small amount of data, and it is almost impossible to utilize for a large amount of image data.

Therefore, it is necessary to study an algorithm for the automatic recognition of habitat elements from bird images, as this will be of great help for ornithology research. The automatic identification of habitat elements can be regarded as an image classification problem. Given the extraordinary performance of DCNNs in image classification problems, we assume that the use of this technology to identify habitat factors from bird images is also feasible and effective.

To our knowledge, this study is the first attempt to identify habitat elements in bird images and to build a database for this kind of research. In summary, the contributions of this article to the field mainly include: We built a dataset, Habitat Elements of Bird Images (HEOBs-10), for identifying habitat elements from bird images; HEOBs-10 contains 2135 images across 10 categories. We used four popular DCNNs to implement automatic identification for habitat elements and achieved good results, which verified the effectiveness of DCNNs in solving the problem of identifying habitat elements from bird images and provided a baseline for future research.

## 2. Materials and Methods

### 2.1. Data Acquisition

Since no public image dataset exists for identifying habitat elements, it was necessary to build an appropriate dataset. For this reason, we built a database called HEOBs-10. The database contains 10 categories, each with approximately 200 images; all images in the database are randomly divided into three parts at a ratio of 3:1:1 (for the training set, the validation set, and the test set), and the distribution of samples for each subset tends to be balanced (Table 1). The training set data are used to train the developed models; the validation set data are used to monitor the training process, which decides when to stop training and find the best model; and the test set data are only used to evaluate the performance of the obtained model and cannot be used for model training.

**Figure 1:**
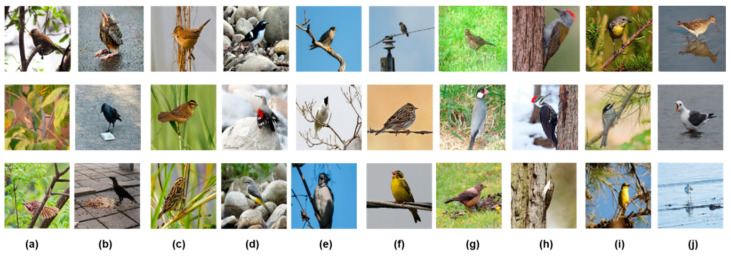
Sample images from our proposed database, HEOBs-10. Ten habitat elements are included. The three images in each column belong to the same category, and the letters a to j refer to broad leaves, hard ground, stalk, stone, leafless, wire, grassland, trunk, coniferous tree, and water, respectively.

The majority of the images in the database were mainly contributed by the citizen science project called BirdFans in China [36] and before the start of this study, our team had obtained approximately 20,000 bird images from BirdFans in China for use in bird image analysis. First, we determined the tags that may be used for the identification of habitat elements. This process was completed by looking up related literature, consulting bird researchers, and quickly browsing existing images. Combining actual needs and existing image data, we initially selected 17 alternate labels (such as water, sky, broad leaves, etc.) that were used as habitat element category labels. Then, we used the labels to create corresponding category folders and manually classified the abovementioned images. During the classification process, images with clear categories were preferentially selected, and images whose habitat elements were difficult to identify due to blurred backgrounds were removed. Then, we checked and removed duplicate and unreadable images. This process was automatically completed by scripting in Python. The number of images used for model training is a key component of the development of a quality assurance process [37]. Some categories with fewer than 200 samples were not included in the database. After completing the above steps, we obtained a dataset containing 10 categories. We also collected some images from Macaulay Library at the Cornell Lab of Ornithology and eBird [38] as a supplement, and these new images were used to replace some different but similar images that may have been caused by continuous shooting. This processing step can increase the diversity of a single data category, which is beneficial for the stability of the proposed algorithm [39]. Very few pictures contained two or more habitat elements, and we only used the most significant category as the true label in such cases. Figure 1 shows some of the samples in the database.

### 2.2. DCNN Models

**Figure 2:**
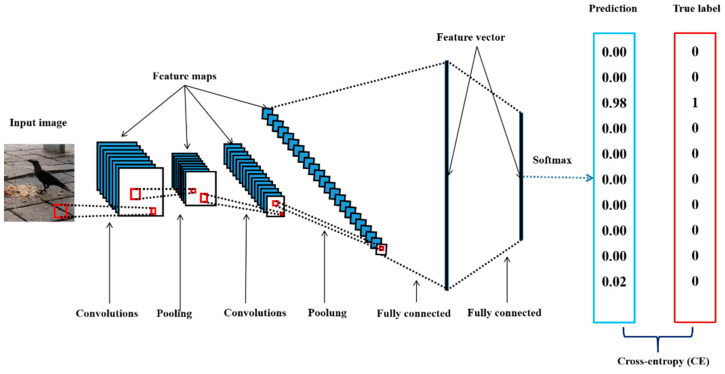
Overview of the deep convolutional neural network (DCNN) architecture. A DCNN consists of several layers (including but not limited to a convolutional layer, pooling layer, and fully connected layer) of abstraction that tend to gradually convert raw data into more abstract concepts [42]. For example, the raw pixels of the input data are first transformed into low-level features, then more complex features, and then high-level features until a final prediction is made by the final fully connected layer, which employs a softmax function that can return a vector of the same size as the number of categories to be identified. Each element of the vector is a value between 0 and 1, with higher values signalling higher confidence of the model in the predicted category of the input image.

Deep convolutional neural networks are very similar to artificial neural networks (ANNs) [40], which are composed of large numbers of neurons with learnable weights and biases. Typically, these neurons are aggregated into layers. A typical DCNN consists of a sequence of layers, and every layer of the network transforms one volume of activations into another through an activation function. Three main types of layers (convolutional layers, pooling layers, and fully connected layers) are used to build DCNN architectures. Note that convolutional layers and fully connected layers contain learnable parameters (the weights and biases of the neurons). For image classification tasks, during the training phase, when a DCNN receives input data, it produces a prediction through forward propagation [15]. The prediction is usually interpreted as the probability distribution of the categories predicted by the model, and a higher value in the probability distribution usually indicates that the DCNN is more confident that the image belongs to the corresponding category (Figure 2). The distance between the predicted probability distribution and the one-hot encoding-based [41] representation of the data label is recorded as a loss. Then, the network adjusts its parameters through backpropagation [15] to minimize this loss. Backpropagation is usually implemented by the gradient descent method [15].

In image classification tasks, cross entropy (CE) [43] is often used to calculate the loss. The CE indicates the distance between what the model believes the output distribution should be and what the target distribution is [43]. We use P and Q to represent the output vector of the model predictions and the target vector composed of the true labels, respectively.

The cross-entropy H$\left(p,q\right)$ of the two probability distributions P and Q obeys the following system of equations:

(1) $$P=\;\left\{p\left(x_{1}\right),\;p\left(x_{2}\right),p\left(x_{i}\right),\ldots ,p\left(x_{n}\right)\right\},$$

(2) $$Q=\;\left\{q\left(x_{1}\right),q\left(x_{2}\right),q\left(x_{i}\right),\ldots ,q\left(x_{n}\right)\right\},$$

(3) $$\mathrm{CE}=H\left(p,q\right)\;=-\overset{n}{\underset{1}{\sum }}p\left(x_{i}\right)\;\times \;q\left(x_{i}\right),$$

where i represents the index of the output vector component (or the categories in the target vector), and *n* represents the number of categories.

AlexNet [44] won the 2012 ImageNet Large Scale Visual Recognition Challenge, a benchmark in object category classification with millions of images, with a significant advantage. Since then, algorithms based on DCNNs have been widely used in various computer vision tasks. DCNNs not only have outstanding performance in various tasks but also use an end-to-end approach. Manual intervention is greatly reduced, making the applications of DCNNs more convenient; therefore, their application range has been further expanded. After AlexNet was developed, additional new DCNNs with excellent performance were proposed, such as the Visual Geometry Group (VGG) network [45], ResNet [46,47], GoogLeNet [48], and DenseNet [49]. Compared with AlexNet, these new network models increase the number of layers and optimize the structure of the network. For example, VGG replaces the large convolution kernel used by AlexNet with two smaller convolution kernels; ResNet adds a residual structure. These optimizations enable the networks to not only increase their fitting abilities but also obtain significant performance improvements.

A DCNN can also be regarded as being composed of two parts: a feature extractor and a classifier. The feature extractor can extract low-level features, more complex features, and high-level features from the original image to obtain a feature map, which is then expanded into a high-dimensional feature vector. The high-dimensional feature vector is then fed to the classifier, where the vector undergoes some linear transformations and nonlinear transformations, and finally passes through the softmax function; the output is a vector of the same size as the number of categories to be identified.

**Figure A1:**
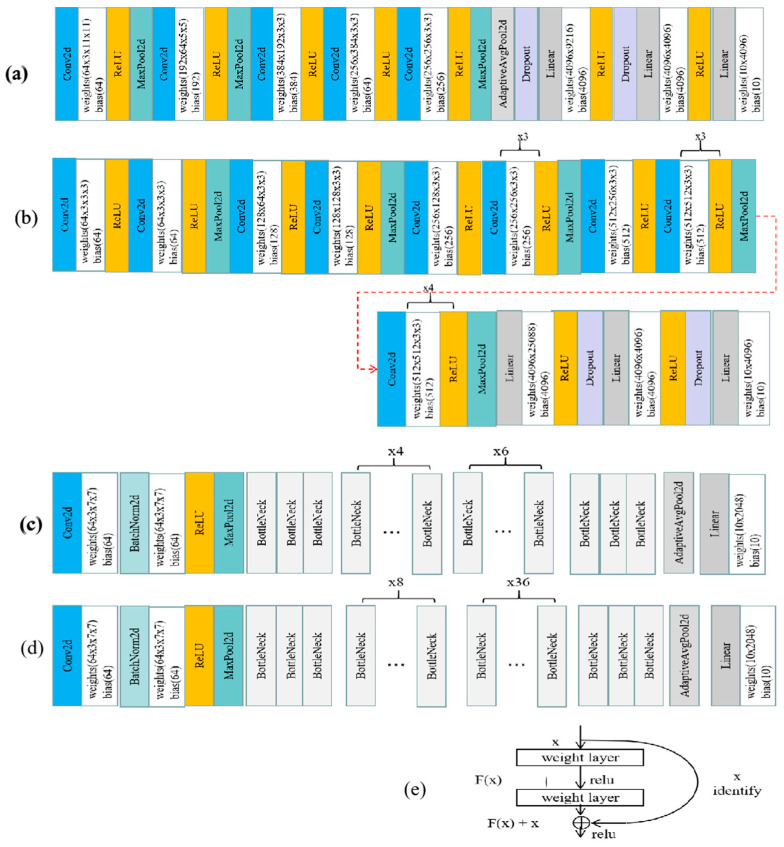
Architecture of the four tested models: (**a**) AlexNet, (**b**) VGG19, (**c**) ResNet50, (**d**) ResNet152 and (**e**) residual structure: the bottleneck in (**c**) and (**d**). In addition to the typical structures of convolutional layers, pooling layers, and fully connected layers, AlexNet also adds Rectified Linear Unit (ReLU) [79] layers and dropout [80] layers. These new structures are one of the reasons why the performance of AlexNet is more prominent than the performance of previous convolutional neural networks. VGG19 replaces the large convolution kernel with a smaller convolution kernel based on AlexNet, which further improves the performance. ResNet50 and ResNet152 go a step further. They use a stacked residual network structure to make the model deeper so that this type of network can obtain improvements without performance degradation.

In this work, we used four DCNN models in the PyTorch [50] model library as our basic networks, including AlexNet and VGG19 [51], and two ResNet series networks, ResNet50 and ResNet152. The architectures of various models are represented in Figure A1.

### 2.3. Transfer Learning

Transfer learning is used to improve a model from one domain by transferring information from a related domain [52] and is widely used in various image classification tasks because it can shorten the required training time, make the model converge faster, and significantly improve the performance of the model when the data size is relatively small. In practice, a model pretrained on ImageNet is often used as the initial network model, and then the structure is modified according to the specific task. Then, the new data are used for training, that is, fine-tuning [53]. This approach has achieved good results in solving a wide range of computer vision problems [54]. DCNNs require a large number of image instances for training; however, in this work, because the data size was not large enough, we adopted the transfer learning method, and the classifier part of the utilized network was modified according to the number of categories in HEOBs-10.

### 2.4. Implementation and Preprocessing

A Linux server with one GV100GL (a Tesla V100 PCIe GPU with 32 GB of memory, Santa Clara, CA, USA) was used to train all the networks. The networks were implemented using Python 3.7.4 and PyTorch (Version 1.3.1, Facebook, Menlo Park, CA, USA) [50]. We used 60% (1268 images) of the dataset for training, 20% (435 images) as verification images, and the remaining 20% as test images; the counts of all habitat element categories are shown in Table 1. The code for the CNN training and prediction method described in this paper is available on GitHub [55].

After the dataset was preprocessed through the method described above, we needed to set the hyperparameters involved in the training process. These hyperparameters have different effects on the model training time, convergence, and equipment load. Therefore, before starting the training process, we employed several pre-experiments and obtained the best combination of hyperparameters using the grid search method [56]. Table 2 summarizes the primary hyperparameters that governed the DCNNs during our experiments.

The learning rate controls how much the model should be changed in response to the loss each time the model’s weights are updated. A large learning rate may cause the model to oscillate during the training process and fail to converge; a learning rate that is too small greatly increases the convergence time. Here, we adopted a policy called step decay, which can adaptively change the learning rate automatically as the training procedure progresses. Given the machine’s capacity and algorithm convergence, usually, an entire dataset (training set, validation set, or test set) is not passed through a model at once. Instead, the complete dataset is passed to the same model iteratively in batches. The batch size refers to the number of training images utilized in one iteration. One epoch represents that the entire dataset is passed forward and backward through the model only once. To obtain a model with good performance, the model needs to be trained for several epochs.

### 2.5. Training Models

We obtained four DCNN models, which were pretrained on approximately 1.28 million images (1000 object categories) from the ImageNet, from PyTorch’s model management library. We modified the models according to the number of categories in our dataset and used each modified model as the initial network model. During the training phase, to increase the size of the training set and decrease overfitting problems [62], multiple image transformations, such as rescaling (all input images were resized to 224 × 224 to follow the model specification); random rotation; random changes in the brightness, contrast, and saturation of an image; random horizontal flip; and center-crop augmentation, were used to train each model. The data augmentation procedure was automatically computed before training.

**Figure 3:**
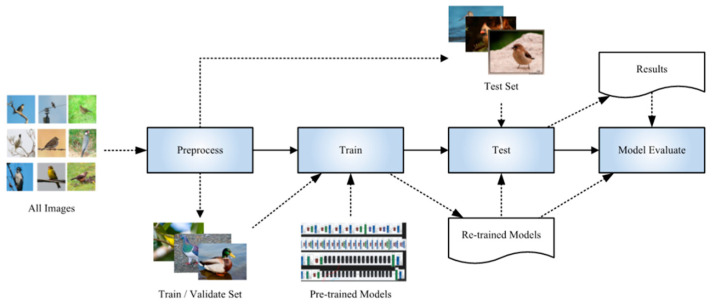
Pipeline of our proposed framework for identifying habitat elements.

The whole experiment in our work was performed in two separate stages. In the first stage, we used the training set and validation set data to fine-tune each pretraining model in turn, recording the training loss and validation accuracy of the model in each epoch. In the training phase, the model with updated parameters after the first epoch was automatically saved as a temporary optimal model. At the end of the new epoch, we compared the verification accuracy of the new model with that of the previous model. If the validation accuracy of the new model was higher than that of the previous model, the new model overwrote the previously saved optimal model. Otherwise, the previous model was retained. This was repeated until the last (50th) epoch was finished. After performing these steps (Figure 3), we obtained 4 retrained DCNN models with optimal validation accuracies.

In the second stage, to observe the effect of the size of the training dataset on the effectiveness of the DCNNs in identifying habitat factors, we specifically processed the initial dataset. We kept the validation set and test set unchanged. We copied the four original training datasets and randomly selected 20%, 40%, 60%, and 80% of the images from these copied datasets to generate four new training datasets. Then, we used these subdatasets of different sizes and the original verification set and test set to repeat the procedure of the first stage.

For deep learning, generally, it is difficult for the same team in the same location to obtain the exact same results on different experiments with the same precision under the same experimental setup, such as the hardware and software settings used during multiple trials [63]. To obtain more reference data, we repeated the two experimental stages 10 times.

### 2.6. Performance Evaluation Indicators

We used the 435 test images to evaluate the retrained models. Note that during the model evaluation stage, each model’s parameters were not updated. All the test images were divided into four categories according to the real labels and the prediction results. There are altogether four basic counts: true positives (TPs), true negatives (TNs), false positives (FPs), and false negatives (FNs). The numbers of correctly predicted test images are indicated by the TPs and TNs, and the numbers of incorrectly predicted images are the FNs and FPs. Based on the above definitions, the following four indicators commonly used to evaluate the performance of classification models can be defined: accuracy, precision, recall, and F1-score.

Accuracy: This metric is defined as the ratio of correctly classified images to the total number of images in the dataset and can be defined as follows:

(4) $$\mathrm{Accuracy}=\frac{TP+TN}{TP+TN+FP+FN}.$$

Precision, Recall, and F1-score: These metrics are used to measure how close the results obtained for each category are to the corresponding real labels. Take the trunk class of test images as an example. The precision refers to the ratio of the number of images correctly predicted as trunks (TPs) to the total number of images predicted as trunks (TP+FP); the recall rate refers to the ratio of the number of images correctly predicted as trunks (TPs) to the number of images with the true label of “Trunk” (TP + FN). The F1-score is computed as the harmonic average of precision and recall. Therefore, the above three indicators can be defined as:

(5) $$\mathrm{Precision}=\frac{TP}{TP+FP},$$

(6) $$\mathrm{Recall}=\frac{TP}{TP+FN},$$

(7) $$F1-\mathrm{score}=\frac{2\times Precision\times Recall}{Precision+Recall}.$$

## 3. Results

**Figure 4:**
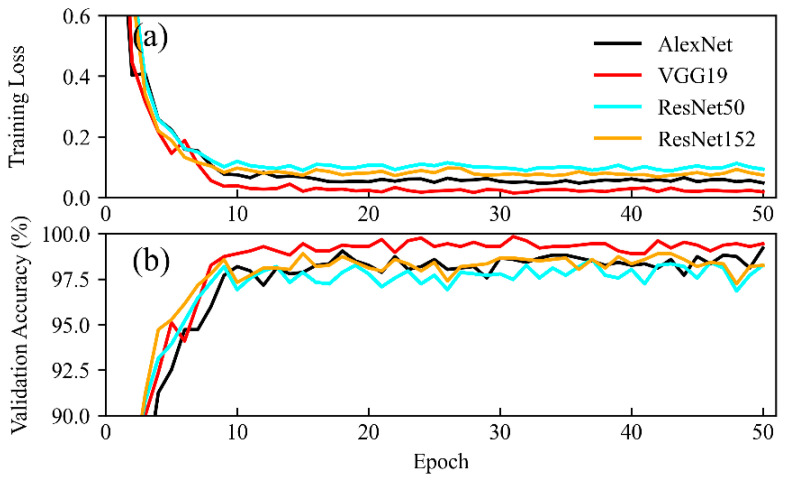
Training losses and accuracies of the tested models. (**a**) For each of the models, as the number of training rounds increased, the loss between the model’s predicted value and the true value showed a decreasing trend. (**b**) At the same time, the model’s accuracy on the validation set showed an upward trend. The models tended to converge in approximately 20 epochs, and the validation accuracy of each model also tended to stabilize, which indicates that the networks were fully trained.

In our experiment, during the training phase, the proposed models tended to converge in approximately 20 epochs, and the verification accuracy of each model also tended to stabilize, which indicates that each network was fully trained (Figure 4).

On the whole, good results were obtained for the proposed models (Table 3). The ResNet152-based model yielded the best test accuracy rate (95.52%). The AlexNet-based model yielded the lowest test accuracy rate (89.48%).

**Figure 5:**
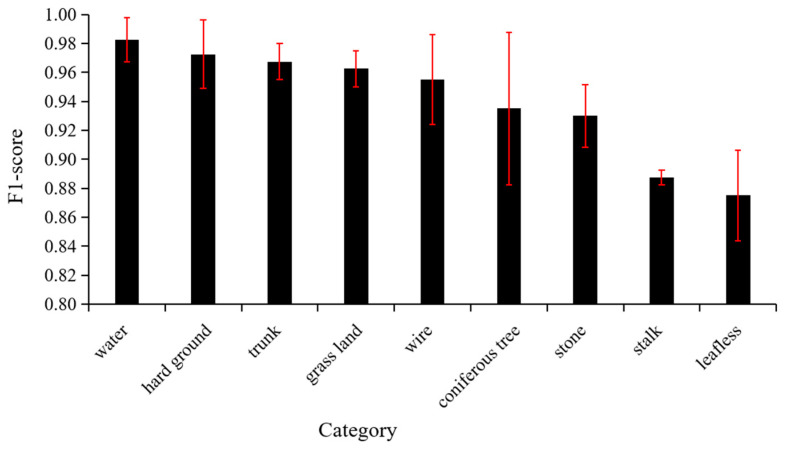
F1-scores of different categories on the test set. Error bars displaying the standard deviations are shown.

The F1-score of each habitat factor is greater than 0.80 (Figure 5, Table A1), which indicates that the models exhibited good performance on the test set. There were significant differences (analysis of variance (ANOVA) *p* < 0.05, Table A2) in the abilities of the models to identify various habitat elements on the test set. This significance is mainly reflected in the fact that the average F1 scores of “leafless” and “stalk” were significantly lower than those of other categories (Table A3), and there were no significant differences for the other habitat elements.

**Figure 6:**
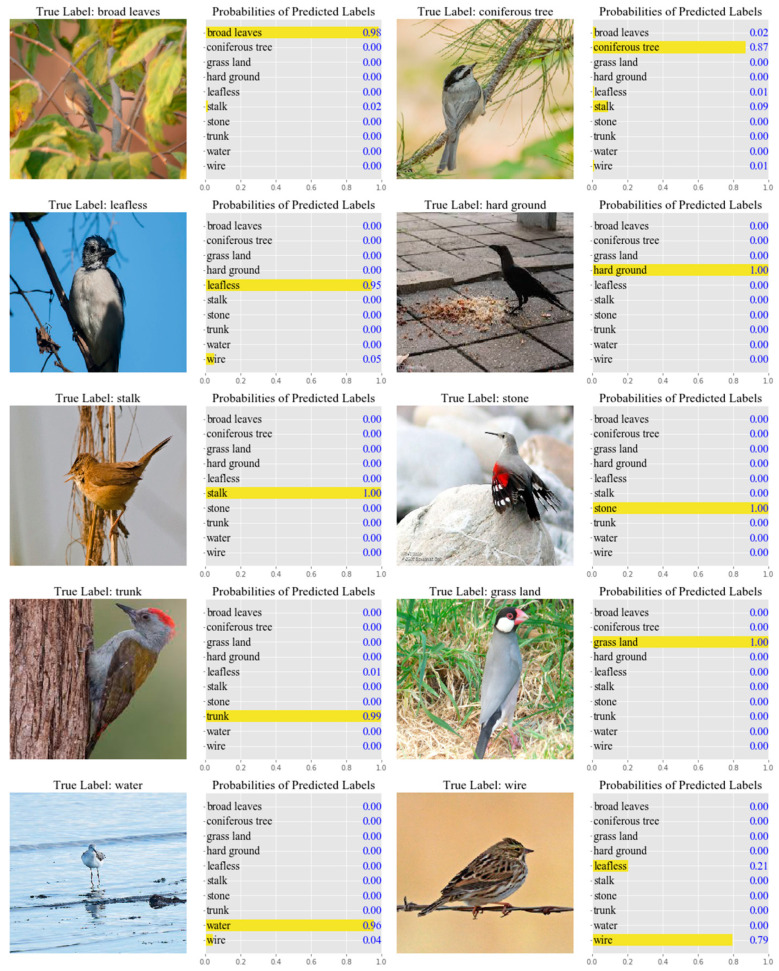
The predictions given by AlexNet for the images in the test set. The real labels and the top 10 predictions are shown. The number beside each label indicates the corresponding probability or prediction confidence. For each image, the sum of the probability values of all its corresponding tags is equal to 1, and this was determined by the activation function used in the proposed algorithm.

We utilized AlexNet to make predictions for 10 samples randomly picked from different categories in the test set. We observed that the model was very confident in the predictions of these sample images, and most of the probability values were above 0.90 (Figure 6).

**Figure 7:**
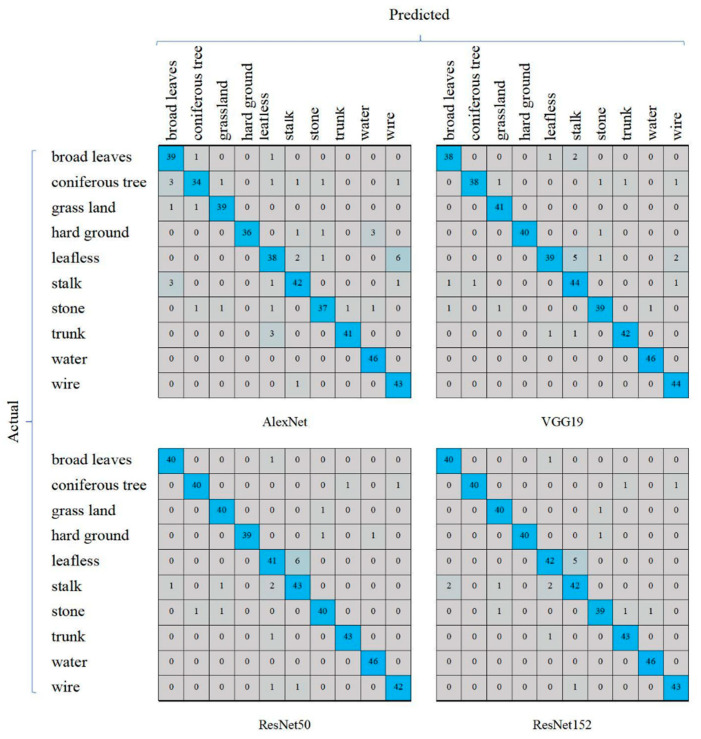
Confusion matrix (or error matrix) comparison among the above models. Taking the confusion matrix of AlexNet as an example, I and J represent the row number and column number of the elements in the matrix, respectively; then, the element (i, j) of each confusion matrix represents the number of predictions of category j given that the actual label was class i, with i and j referring to the classes from the category names at the left and top of the figure. Note that AlexNet recognized “leafless” as “wire” most times, while the other three networks did not. However, the other three networks confused “leafless” and “stalk” with each other.

**Figure 8:**
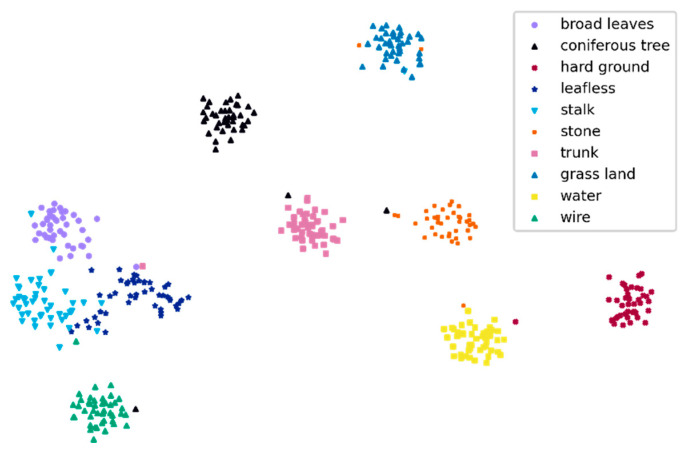
T-distributed stochastic neighbour embedding (T-SNE) [64] visualization of the last hidden layer representations produced by ResNet152 for ten habitat element classes (the coloured point clouds represent the different habitat element categories, showing how the algorithm clustered these categories). Most habitat elements were grouped independently according to their categories, but broad-leaved trees, leafless plants, and stalk were more concentrated, and wires were closer to these three categories.

We observed that the four DCNN models performed well in terms of the recognition of water, and no errors occurred. When identifying “leafless” and “stalk”, the performances were relatively poor; this was consistent with the F1-score evaluation (Figure 5). For “leafless” recognition, the numbers of errors were not less than 5; for “stalk recognition”, the numbers of errors were greater than or equal to 4. ResNet152 misidentified 5 leafless sheets as stalks and misidentified 2 sheets of stalks as leafless images, indicating that ResNet152 was easily confused by “leafless” and “stalk” (Figure 7); this was consistent with the T-SNE visualization results (Figure 8).

**Figure 9:**
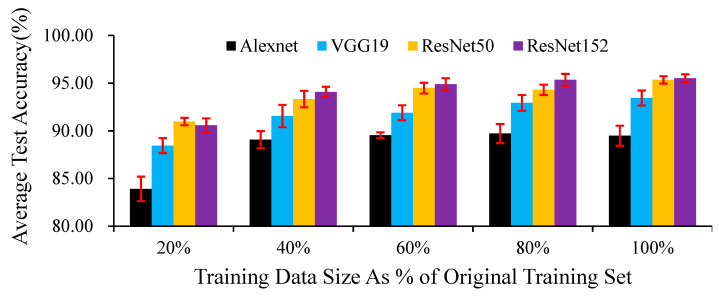
Average test accuracies of the proposed models trained with training sizes ranging from 20% to 100% of the images in the original training set. Error bars displaying the standard deviations within 50 epochs are shown.

Although DCNNs have higher requirements than other networks regarding the number of samples, in our research, when using 20% of the training set data, the test accuracies of the four networks exceeded 0.80 (Figure 9). In our experiments, the impact of increasing the training set size on different networks is not consistent. For AlexNet (Table A4), using 20% of the images in the original training set as the sub-training data set, the test accuracy was significantly lower than that of other larger training data sets; as the training set size increased, the test accuracy rate did not show a significant change. For VGG19 (Table A5) and ResNet152 (Table A6), when the training set size is increased to 60% of the initial training set size, the test accuracy is significantly improved; but when a larger training set is used to train the model, the test accuracy does not change significantly. For ResNet50 (Table A7), as the training set increased, the test accuracy showed a significant improvement trend, but there was no significant difference between the 60% and 80% training sets size.

## 4. Discussion

As expected, our study verified the feasibility and effectiveness of using DCNNs to automatically identify habitat elements, and the best accuracy rate reached 97.76%. Although we only used ten habitat elements as the objects of recognition in our research, DCNNs have also achieved good results in terms of the recognition of 1000 categories in the ImageNet recognition task [65], so we have reason to believe that if more categories of images are provided, our method can also identify more habitat elements.

In our work, when the four network models recognized “leafless” and “stalk”, the recognition rates were relatively low, and the two categories were prone to being confused with one another. However, the training images of these two habitat factors were not the least common. This result may have been caused by the visual similarity of the two types of habitat elements.

We can identify habitat elements from bird images, which will help us to understand the characteristics of bird habitats and a given bird’s preferences for habitat elements by analysing the object co-occurrence [66] relationships in each image. However, it should be noted here that it is not sufficient to infer the habitat types of birds based on the identified habitat elements alone. Taking an image containing water as an example, without the support of more information, it cannot be inferred that the habitat is in a puddle, lake, or sea, and it is not even easy to judge whether the image is a country or town scene. On the one hand, some different habitats share the same habitat elements, such as water; on the other hand, the identification of the habitat category itself may also be disturbed by shooting elements, such as the camera view angles and depth of field. In terms of judging habitat types, we can obtain more reliable and richer information based on identifying habitat elements and combining image metadata [67], such as global positioning system (GPS) data and shooting time data, and this will be a worthwhile approach.

The continuous in-depth research and application of image recognition technology in various fields have also caused some concerns among researchers [68,69,70,71] about their personal data being parsed; this kind of worry should be paid attention to and taken seriously. Some image datasets used for species identification, such as the Snapshot Serengeti dataset [72], may inadvertently capture faces or other pictures containing personal information during the image collection process. If they are not processed, there will be a risk of privacy leakage. However, this risk is not uncontrollable. From a technical point of view, if the data containing personal information are filtered out during the model training phase or the use of personal information as a category label is avoided, the model recognition range can be limited to a predetermined target category. In our research, during the processes of data collection and labelling, we did not identify categories that had little to do with the identification of habitat factors as identification objects and screened out images of human faces and hands. For the development of related software or applications, a qualified professional department can review and record the code provided by the developer to clarify the scope of identification. Different types of users should be given different permissions. For example, for some closed/protected areas or sanctuaries, supervision is costly and difficult. Image analysis technology can be used to monitor illegal hunting and poachers. However, for ordinary users, functions involving face recognition or human behaviour analysis should be strictly controlled.

We used a single-label method to annotate the images in our experiments; this approach could not fully express the information about the habitat elements in the images, although most images in the dataset could be processed in this way. In addition, our algorithm is invalid for unfamiliar data (the real labels of which are not included in our dataset); this fact is caused by the activation function used by the algorithm. Therefore, for future research, we believe that the use of multi-label learning [73,74] methods can compensate for these deficiencies. In addition, due to time and human factors, we only identified 10 habitat elements, which are far less than actual needs. We hope that more collaborators can try to identify more types of habitat elements, as this will be meaningful work for animal ecology and zoology research.

Mining interesting knowledge from bird image data is helpful for promoting bird research. Tryjanowski et al. [75] used YouTube video resources to study the interesting phenomenon of alcohol ingestion by birds; Stoddard et al. [76] used image processing technology to study which features of eggs and the background substrate may be effective in preventing predator detection. The combination of bird species recognition, habitat recognition, and bird behaviour recognition will facilitate such research. This will make it possible to automatically understand bird images, allowing researchers to save considerable data processing time and spend more time and energy thinking and discovering new knowledge.

In general, the promising performance of this line of research provides us confidence that DCNNs can be used to automatically identify habitat elements from bird images. The practical application of this technology will promote research on the relationships between birds and habitat elements. At the same time, this technology may also contribute to improving the accuracy of bird recognition because some studies [77,78] have shown that image background information can improve target recognition accuracy.

## 5. Conclusions

In the present study, we demonstrated the effectiveness of DCNNs in automatically identifying habitat elements from bird images. For the needs of research, we established a dataset called HEOBs-10, which is composed of bird images related to 10 categories of habitat elements. Good results were obtained from all the proposed models. ResNet-152-based models yielded the best validation accuracy rate (95.52%); the AlexNet-based model yielded the lowest test accuracy rate (89.48%). The set of experiments performed in this work provides baseline results for the introduced database, which may minimize the lack of a robust public dataset in the field of automatically identifying habitat elements in bird images, thereby making it possible to conduct future benchmarks and evaluations. An evaluation of the performance of the proposed dataset in the real world requires further research. First, the dataset needs to be supplemented by additional bird images containing new categories of habitat elements. Second, the dataset should encourage the use of multilabel learning methods to identify habitat elements, as such methods will be more suitable for the needs of real scenarios than the single-label method. Third, DCNNs can be used to establish relationship graphs between birds themselves and habitat elements, which will be a meaningful exercise.

## Figures and Tables

**Table 1:** Partitions of the 10 categories in the HEOBS-10 dataset.

Categories	Training Set	Validation Set	Test Set
Broad leaves	118	41	41
Coniferous tree	122	42	42
Hard ground	118	41	41
Leafless	138	47	47
Stalk	139	47	47
Stone	124	42	42
Trunk	131	44	44
Grassland	118	41	41
Water	133	46	46
Wire	127	44	44
Total	1268	435	435

**Table 2:** Hyperparameters used in our experiments.

Hyperparameters	Values
Initial learning rate [57]	0.001
Optimizer [58]	stochastic gradient descent (SGD) [59]
Learning rate policy [57]	step decay [60] (momentum = 0.9; step size = 7; gamma = 0.1)
Batch size [57]	32
Number of epochs [61]	50

**Table 3:** Classification accuracies of various models on the test set.

Model Name	Validation Accuracy (Mean ± SD%)	Test Accuracy (Mean ± SD%)
AlexNet	91.11 ± 0.54	89.48 ± 1.05
VGG19	96.05 ± 0.59	93.45 ± 0.79
ResNet50	97.16 ± 0.38	95.34 ± 0.39
ResNet152	97.76 ± 0.36	95.52 ± 0.40

## Data Availability

Since some of the images in the dataset proposed in this study are from eBird, the public release of our dataset conflicts with the data usage licence of the website, and we have not been authorized to do so, so the download link for the dataset cannot be provided here. However, the bird images used in the research are available from the websites of eBird (https://ebird.org/media/catalog, accessed on 25 July 2020) and BirdFans (https://www.birdfans.com, accessed on 15 June 2019).

## Appendix A

**Table A1:** Metrics of various models.

Model	Class	Precision	Recall	f1-Score
Alexnet	broad leaves	0.85	0.95	0.90
	coniferous tree	0.92	0.81	0.86
	grass land	0.95	0.95	0.95
	hard ground	1.00	0.88	0.94
	leafless	0.84	0.81	0.83
	stalk	0.89	0.89	0.89
	stone	0.93	0.88	0.90
	trunk	0.98	0.93	0.95
	water	0.92	1.00	0.96
	wire	0.84	0.98	0.91
VGG19	broad leaves	0.95	0.93	0.94
	coniferous tree	0.97	0.90	0.94
	grass land	0.95	1.00	0.98
	hard ground	1.00	0.98	0.99
	leafless	0.95	0.83	0.89
	stalk	0.85	0.94	0.89
	stone	0.93	0.93	0.93
	trunk	0.98	0.95	0.97
	water	0.98	1.00	0.99
	wire	0.92	1.00	0.96
ResNet50	broad leaves	0.98	0.98	0.98
	coniferous tree	0.98	0.95	0.96
	grass land	0.95	0.98	0.96
	hard ground	1.00	0.95	0.97
	leafless	0.89	0.87	0.88
	stalk	0.86	0.91	0.89
	stone	0.95	0.95	0.95
	trunk	0.98	0.98	0.98
	water	0.98	1.00	0.99
	wire	0.98	0.95	0.97
ResNet152	broad leaves	0.95	0.98	0.96
	coniferous tree	1.00	0.95	0.98
	grass land	0.95	0.98	0.96
	hard ground	1.00	0.98	0.99
	leafless	0.91	0.89	0.90
	stalk	0.88	0.89	0.88
	stone	0.95	0.93	0.94
	trunk	0.96	0.98	0.97
	water	0.98	1.00	0.99
	wire	0.98	0.98	0.98

**Table A2:** ANOVA results regarding the F1-scores of various categories.

Sources	SS	df	MS	F	*p*-Value	Eta-sq	RMSSE	Omega Sq
Between Groups	0.0459	9.0000	0.0051	6.8324	*p* < 0.001	0.6721	1.3069	0.5675
Within Groups	0.0224	30.0000	0.0007					
Total	0.0682	39.0000	0.0017					

**Table A3:** Tukey’s HSD Post Hoc Test results regarding the F1-score of various categories.

Group 1	Group 2	Mean	Std Err	q-Stat	Lower	Upper	*p*-Value	Mean-Crit	Cohen d
broad leaves	coniferous tree	0.0100	0.0137	0.7323	(0.0559)	0.0759	0.9999	0.0659	0.3662
broad leaves	grass land	0.0175	0.0137	1.2816	(0.0484)	0.0834	0.9951	0.0659	0.6408
broad leaves	hard ground	0.0275	0.0137	2.0139	(0.0384)	0.0934	0.9098	0.0659	1.0070
broad leaves	stone	0.0150	0.0137	1.0985	(0.0509)	0.0809	0.9984	0.0659	0.5493
broad leaves	trunk	0.0225	0.0137	1.6478	(0.0434)	0.0884	0.9723	0.0659	0.8239
broad leaves	water	0.0375	0.0137	2.7463	(0.0284)	0.1034	0.6426	0.0659	1.3731
broad leaves	wire	0.0100	0.0137	0.7323	(0.0559)	0.0759	0.9999	0.0659	0.3662
broad leaves	leafless	0.0700	0.0137	5.1263	0.0041	0.1359	**0.0303**	0.0659	2.5632
broad leaves	stalk	0.0575	0.0137	4.2109	(0.0084)	0.1234	0.1282	0.0659	2.1055
coniferous tree	grass land	0.0275	0.0137	2.0139	(0.0384)	0.0934	0.9098	0.0659	1.0070
coniferous tree	hard ground	0.0375	0.0137	2.7463	(0.0284)	0.1034	0.6426	0.0659	1.3731
coniferous tree	stone	0.0050	0.0137	0.3662	(0.0609)	0.0709	1.0000	0.0659	0.1831
coniferous tree	trunk	0.0325	0.0137	2.3801	(0.0334)	0.0984	0.7960	0.0659	1.1900
coniferous tree	water	0.0475	0.0137	3.4786	(0.0184)	0.1134	0.3288	0.0659	1.7393
coniferous tree	wire	0.0200	0.0137	1.4647	(0.0459)	0.0859	0.9873	0.0659	0.7323
coniferous tree	leafless	0.0600	0.0137	4.3940	(0.0059)	0.1259	0.0980	0.0659	2.1970
coniferous tree	stalk	0.0475	0.0137	3.4786	(0.0184)	0.1134	0.3288	0.0659	1.7393
grass land	hard ground	0.0100	0.0137	0.7323	(0.0559)	0.0759	0.9999	0.0659	0.3662
grass land	stone	0.0325	0.0137	2.3801	(0.0334)	0.0984	0.7960	0.0659	1.1900
grass land	trunk	0.0050	0.0137	0.3662	(0.0609)	0.0709	1.0000	0.0659	0.1831
grass land	water	0.0200	0.0137	1.4647	(0.0459)	0.0859	0.9873	0.0659	0.7323
grass land	wire	0.0075	0.0137	0.5493	(0.0584)	0.0734	1.0000	0.0659	0.2746
grass land	leafless	0.0875	0.0137	6.4079	0.0216	0.1534	**0.0030**	0.0659	3.2040
grass land	stalk	0.0750	0.0137	5.4925	0.0091	0.1409	**0.0160**	0.0659	2.7463
hard ground	stone	0.0425	0.0137	3.1124	(0.0234)	0.1084	0.4777	0.0659	1.5562
hard ground	trunk	0.0050	0.0137	0.3662	(0.0609)	0.0709	1.0000	0.0659	0.1831
hard ground	water	0.0100	0.0137	0.7323	(0.0559)	0.0759	0.9999	0.0659	0.3662
hard ground	wire	0.0175	0.0137	1.2816	(0.0484)	0.0834	0.9951	0.0659	0.6408
hard ground	leafless	0.0975	0.0137	7.1403	0.0316	0.1634	**0.0007**	0.0659	3.5701
hard ground	stalk	0.0850	0.0137	6.2248	0.0191	0.1509	**0.0042**	0.0659	3.1124
stone	trunk	0.0375	0.0137	2.7463	(0.0284)	0.1034	0.6426	0.0659	1.3731
stone	water	0.0525	0.0137	3.8448	(0.0134)	0.1184	0.2114	0.0659	1.9224
stone	wire	0.0250	0.0137	1.8308	(0.0409)	0.0909	0.9473	0.0659	0.9154
stone	leafless	0.0550	0.0137	4.0278	(0.0109)	0.1209	0.1657	0.0659	2.0139
stone	stalk	0.0425	0.0137	3.1124	(0.0234)	0.1084	0.4777	0.0659	1.5562
trunk	water	0.0150	0.0137	1.0985	(0.0509)	0.0809	0.9984	0.0659	0.5493
trunk	wire	0.0125	0.0137	0.9154	(0.0534)	0.0784	0.9996	0.0659	0.4577
trunk	leafless	0.0925	0.0137	6.7741	0.0266	0.1584	**0.0015**	0.0659	3.3870
trunk	stalk	0.0800	0.0137	5.8587	0.0141	0.1459	**0.0083**	0.0659	2.9293
water	wire	0.0275	0.0137	2.0139	(0.0384)	0.0934	0.9098	0.0659	1.0070
water	leafless	0.1075	0.0137	7.8726	0.0416	0.1734	**0.0002**	0.0659	3.9363
water	stalk	0.0950	0.0137	6.9572	0.0291	0.1609	**0.0011**	0.0659	3.4786
wire	leafless	0.0800	0.0137	5.8587	0.0141	0.1459	**0.0083**	0.0659	2.9293
wire	stalk	0.0675	0.0137	4.9433	0.0016	0.1334	**0.0411**	0.0659	2.4716
leafless	stalk	0.0125	0.0137	0.9154	(0.0534)	0.0784	0.9996	0.0659	0.4577

The *p* values in bold font represent significant differences between groups (*p* < 0.05). The *p* values indicate that the average F1-score between “leafless” and “stalk” is significantly lower than those of other categories, and there are no significant differences among the other habitat elements.

**Table A4:** Tukey’s HSD Post Hoc Test results regarding AlexNet’s test accuracies of various training set sizes.

Group 1	Group 2	Mean	Std Err	q-Stat	Lower	Upper	*p*-Value	Mean-Crit	Cohen d
s1	s2	4.9952	0.2703	18.4796	3.9090	6.0814	***p* < 0.001**	1.0862	5.8438
s1	s3	5.4183	0.2703	20.0452	4.3322	6.5045	***p* < 0.001**	1.0862	6.3388
s1	s4	5.2564	0.2703	19.4460	4.1702	6.3426	***p* < 0.001**	1.0862	6.1494
s1	s5	5.2465	0.2703	19.4093	4.1603	6.3327	***p* < 0.001**	1.0862	6.1378
s2	s3	0.4232	0.2703	1.5655	(0.6630)	1.5094	0.8020	1.0862	0.4951
s2	s4	0.2612	0.2703	0.9664	(0.8250)	1.3474	0.9591	1.0862	0.3056
s2	s5	0.2513	0.2703	0.9297	(0.8349)	1.3375	0.9643	1.0862	0.2940
s3	s4	0.1620	0.2703	0.5992	(0.9242)	1.2481	0.9930	1.0862	0.1895
s3	s5	0.1719	0.2703	0.6358	(0.9143)	1.2581	0.9913	1.0862	0.2011
s4	s5	0.0099	0.2703	0.0367	(1.0763)	1.0961	1.0000	1.0862	0.0116

The *p* values in bold font represent significant differences between groups (*p* < 0.05). S1, s2, s3, s4, and s5 respectively refer to the sub-training set composed of 20%, 40%, 60%, 80%, and 100% of the initial training set images. The *p* values indicate that using 20% of the images in the original training set as the sub-training data set, the test accuracy of AlexNet was significantly lower than that on other larger training data sets; as the increased training set size, the test accuracy rate did not show a significant change.

**Table A5:** Tukey’s HSD Post Hoc Test results regarding VGG19′s test accuracies of various training set sizes.

Group 1	Group 2	Mean	Std Err	q-Stat	Lower	Upper	*p*-Value	Mean-Crit	Cohen d
s1	s2	2.4316	0.3067	7.9272	1.1990	3.6642	***p* < 0.001**	1.2326	2.5068
s1	s3	3.9287	0.3067	12.8078	2.6961	5.1612	***p* < 0.001**	1.2326	4.0502
s1	s4	4.5214	0.3067	14.7402	3.2888	5.7540	***p* < 0.001**	1.2326	4.6613
s1	s5	5.0723	0.3067	16.5362	3.8397	6.3049	***p* < 0.001**	1.2326	5.2292
s2	s3	1.4971	0.3067	4.8806	0.2645	2.7296	**0.0102**	1.2326	1.5434
s2	s4	2.0898	0.3067	6.8130	0.8572	3.3224	**0.0002**	1.2326	2.1545
s2	s5	2.6407	0.3067	8.6090	1.4082	3.8733	***p* < 0.001**	1.2326	2.7224
s3	s4	0.5928	0.3067	1.9325	(0.6398)	1.8253	0.6518	1.2326	0.6111
s3	s5	1.1437	0.3067	3.7285	(0.0889)	2.3763	0.0806	1.2326	1.1790
s4	s5	0.5509	0.3067	1.7960	(0.6817)	1.7835	0.7106	1.2326	0.5680

The *p* values in bold font represent significant differences between groups (*p* < 0.05). S1, s2, s3, s4, and s5 respectively refer to the sub-training set composed of 20%, 40%, 60%, 80%, and 100% of the initial training set images. The *p* values indicate that, for VGG19, when the training set size is increased to 60% of the initial training set size, the test accuracy is significantly improved; but after a larger training set is used to train the model, the test accuracy does not change significantly.

**Table A6:** Tukey’s HSD Post Hoc Test results regarding ResNet50′s test accuracies of various training set sizes.

Group 1	Group 2	Mean	Std Err	q-Stat	Lower	Upper	*p*-Value	Mean-Crit	Cohen d
s1	s2	1.9267	0.1455	13.2459	1.3422	2.5112	***p* < 0.001**	0.5845	4.1887
s1	s3	3.3105	0.1455	22.7592	2.7260	3.8950	***p* < 0.001**	0.5845	7.1971
s1	s4	3.1395	0.1455	21.5833	2.5550	3.7240	***p* < 0.001**	0.5845	6.8252
s1	s5	4.2655	0.1455	29.3245	3.6810	4.8500	***p* < 0.001**	0.5845	9.2732
s2	s3	1.3838	0.1455	9.5133	0.7993	1.9683	***p* < 0.001**	0.5845	3.0084
s2	s4	1.2128	0.1455	8.3374	0.6282	1.7973	***p* < 0.001**	0.5845	2.6365
s2	s5	2.3388	0.1455	16.0786	1.7543	2.9233	***p* < 0.001**	0.5845	5.0845
s3	s4	0.1710	0.1455	1.1759	(0.4135)	0.7555	0.9194	0.5845	0.3719
s3	s5	0.9550	0.1455	6.5653	0.3705	1.5395	**0.0003**	0.5845	2.0761
s4	s5	1.1260	0.1455	7.7412	0.5415	1.7105	***p* < 0.001**	0.5845	2.4480

The *p* values in bold font represent significant differences between groups (*p* < 0.05). S1, s2, s3, s4, and s5 respectively refer to the sub-training set composed of 20%, 40%, 60%, 80%, and 100% of the initial training set images. The *p* values indicate that as the training set increased, the test accuracy of ResNet50 showed a significant improvement trend, but there was no significant difference between the 60% and 80% training sets size.

**Table A7:** Tukey’s HSD Post Hoc Test results regarding ResNet152′s test accuracies of various training set sizes.

Group 1	Group 2	Mean	Std Err	q-Stat	Lower	Upper	*p*-Value	Mean-Crit	Cohen d
s1	s2	3.4617	0.2011	17.2106	2.6535	4.2699	***p* < 0.001**	0.8082	5.4425
s1	s3	4.3855	0.2011	21.8033	3.5772	5.1937	***p* < 0.001**	0.8082	6.8948
s1	s4	4.8908	0.2011	24.3156	4.0826	5.6990	***p* < 0.001**	0.8082	7.6893
s1	s5	5.0555	0.2011	25.1344	4.2472	5.8637	***p* < 0.001**	0.8082	7.9482
s2	s3	0.9238	0.2011	4.5927	0.1155	1.7320	**0.0178**	0.8082	1.4523
s2	s4	1.4291	0.2011	7.1051	0.6209	2.2373	**0.0001**	0.8082	2.2468
s2	s5	1.5938	0.2011	7.9238	0.7855	2.4020	***p* < 0.001**	0.8082	2.5057
s3	s4	0.5053	0.2011	2.5124	(0.3029)	1.3136	0.3996	0.8082	0.7945
s3	s5	0.6700	0.2011	3.3311	(0.1382)	1.4783	0.1467	0.8082	1.0534
s4	s5	0.1647	0.2011	0.8188	(0.6436)	0.9729	0.9775	0.8082	0.2589

The *p* values in bold font represent significant differences between groups (*p* < 0.05). S1, s2, s3, s4, and s5 respectively refer to the sub-training set composed of 20%, 40%, 60%, 80%, and 100% of the initial training set images. The *p* values indicate that, for ResNet152, when the training set size is increased to 60% of the initial training set size, the test accuracy is significantly improved; but when a larger training set is used to train the model, the test accuracy does not change significantly.
